# Adults with higher social anxiety show avoidant gaze behaviour in a real-world social setting: A mobile eye tracking study

**DOI:** 10.1371/journal.pone.0259007

**Published:** 2021-10-25

**Authors:** Irma Konovalova, Jastine V. Antolin, Helen Bolderston, Nicola J. Gregory

**Affiliations:** 1 Department of Psychology, Faculty of Science and Technology, Bournemouth University, Poole, Dorset, United Kingdom; 2 School of Psychology, University of Southampton, Southampton, Hampshire, United Kingdom; SWPS University of Social Sciences and Humanities, POLAND

## Abstract

Attentional biases are a core characteristic of social anxiety (SA). However, research has yielded conflicting findings and failed to investigate these biases in real, face-to-face social situations. Therefore, this study examined attentional biases in SA by measuring participants’ eye gaze within a novel eye-tracking paradigm during a real-life social situation. Student participants (N = 30) took part in what they thought was a visual search study, when a confederate posing as another participant entered the room. Whilst all participants avoided looking at the confederate, those with higher SA fixated for a shorter duration during their first fixation on him, and executed fewer fixations and saccades overall as well as exhibiting a shorter scanpath. These findings are indicative of additional avoidance in the higher SA participants. In contrast to previous experimental work, we found no evidence of social hypervigilance or hyperscanning in high SA individuals. The results indicate that in unstructured social settings, avoidance rather than vigilance predominates, especially in those with higher SA.

## Introduction

Social anxiety disorder (SAD) is characterised by an excessive fear of negative social evaluation and avoidance of social situations [1]. People with SAD place great importance on being accepted by others [2]. Paradoxically, by viewing others as fundamentally threatening, those with the disorder (and those with high levels of undiagnosed social anxiety, henceforth referred to as SA) increase the likelihood of being negatively evaluated by others due to their cognitive and behavioural responses to this perceived social threat.

Prominent cognitive and behavioural models of SAD [2–4] set out the processes which lead to this fear of the social world. Whilst these models may diverge in their specifics, all have agreed that biases in attention are critical to the development and maintenance of SA. Furthermore, experimental evidence from behavioural and eye tracking research concords with the view that attentional processes are certainly atypical in those with SA [5–8]. However, differences in methodological approaches to answering the question of how attention is altered in SA have created a complex and at times contradictory picture. Particular complexity surrounds the issue of whether attention to others is increased (referred to hypervigilance) or decreased (referred to as avoidance) in this population. The cause of this uncertainty may be the methodological approaches employed by previous researchers [9]. Critically, although social anxiety is situationally dependent on the existence of other human beings, most of the experimental evidence on this topic, which will be outlined in the next section, has been obtained from studies devoid of any authentic social context, meaning it may not extrapolate to the real world.

As such, in this study, we set out to determine how attention allocation is related to social anxiety symptomology *in vivo*. Specifically, using mobile eye tracking, we evaluated whether the eye movement measures of attention biases reported in the experimental literature are also present when people are physically present with a stranger in a potentially interactive social setting.

### Attentional components of social anxiety: Experimental tasks

Several conclusions as to the attentional signature of SA have been reported in the experimental literature. The first is that people with SA show increased selective attention to faces, particularly threatening faces, referred to as hypervigilance.

Hypervigilance has been operationalised in various ways depending on the methodology employed. The dot probe task has been the paradigm of choice for a large proportion of researchers [5, 7], and within this framework hypervigilance has been operationalised as faster manual responses to detect the probe when preceded by a threatening face as opposed to a neutral face in people with higher SA or those with SAD [7, 10, 11]. In similarly designed tasks which have also recorded eye movements, hypervigilance has been operationalised as *faster first fixations* upon threatening faces versus neutral (or neutral faces compared with objects), or *increased proportion of first fixations* to threatening faces (or neutral faces compared to objects) in higher SA individuals and people with diagnosed SAD [5, 6, 12]. Hypervigilance, as the name suggests, appears to be a fast and early processing bias more akin to a reflective shift of attention, which occurs when stimulus presentation is around 500ms (see [5] and [7] for reviews of dot-probe and eye movement research respectively).

Indeed, when stimulus presentations are increased beyond 1000ms, the opposite direction of attention is often reported: increased reaction times/ longer to fixate the probe [13, 14] / reduced proportion of fixations on the probe [8, 15, 16]–in other words: avoidance. From evidence such as this, some researchers propose a vigilance-avoidance hypothesis of attentional bias in SA, with hypervigilance occurring initially to be replaced by avoidance over longer time periods [11, 17, 18]. This suggests that hypervigilance may be a more reflexive or bottom-up process whereas avoidance is a cognitively driven, slower, top down process.

### Attentional components of social anxiety: Naturalistic tasks

Although this suggestion appears plausible and concords to some degree with the aforementioned models of SAD, it is not known whether the mechanisms highlighted in these trial-based tasks operate in authentic social environments and over longer durations. Some researchers have gone some way to assess attentional bias in SA by recording eye movements of participants whilst they have engaged in less contrived and controlled tasks in real-time although still within the laboratory. Chen, Clarke, MacLeod, Hickie and Guastella [19] found that participants with SAD who gave a speech to an on-screen, pre-recorded audience who gave positive and negative facial feedback at different time points, fixated for longer on non-social regions of the display than non-anxious participants, suggestive of avoidance. Critically however, these authors did not analyse their data for evidence of hypervigilance, (for example by analysing the time taken to fixate on the angry facial expressions after their onset). They did however report evidence of a phenomenon supposedly related to hypervigilance termed *hyperscanning*, seen in the longer overall scanpath executed by the participants with SAD. Hyperscanning had previously been reported in a small number of static face free-viewing studies [20–22]. Hence from this study is was not possible to conclude whether hypervigilance is present in more naturalistic contexts, although avoidance and hyperscanning appeared to be present.

Only one study has examined social attention in SA within a “live”—albeit computer-mediated–context, where it was found that non-clinical participants with higher SA and higher pre-experiment *state* anxiety were shown to display reduced eye -contact throughout a webcam- mediated conversation with a confederate, supporting the avoidance hypothesis [23]. However, no measure of hypervigilance was analysed, hence no conclusions can be drawn about the early processing of social stimuli in a real-time social setting. In addition, the sample size of this correlational study was rather small (N = 20) and as the experiment was highly structured, purposefully interactive and the stimulus pre-selected on the screen, the findings may not extend to a real-life scenario. In sum, these studies focused solely on attentional avoidance whereas hypervigilance did not feature in their rationale, hypotheses or analyses. Although the timescales examined in such real-time tasks are considerably longer than previously outlined experimental, “trail-based “studies (i.e. minutes as opposed to seconds or milliseconds), it is still puzzling that none have examined the initial trial period for evidence of hypervigilance within the eye movement data. As such and, in contrast to the above studies, in our previous work [9] we assessed all three attentional components (hypervigilance, avoidance and hyperscanning) during the passive viewing of a dynamic social scene. We found that high SA individuals looked more at the faces than a group of low SA participants, within the first two-seconds of viewing (a period more akin to those employed in trial-based studies). However, no group differences were found in face viewing or scanpath length over the whole two-minute trial, showing no avoidance or hyperscanning. Although it remains to be examined whether this early bias would extend to a real-life social encounter this is the only study to our knowledge which has analysed all three attentional measures in a naturalistic social task.

### Influence of state anxiety

As demonstrated in the aforementioned study by Howell et al. [23], state anxiety may also play a role in attentional bias in SA. In one eye tracking study investigating the role of induced state anxiety and *non-social* (non-clinical) trait anxiety, it was found that attention to threat, measured by increased viewing time on threatening images was related to state rather than trait anxiety [24]. Further supporting evidence comes from a virtual reality paradigm where a negative relationship was found between state anxiety and amount of gaze directed to a virtual audience in SAD [25]. Therefore, this suggestion that state anxiety may have a role in attentional biases in social anxiety, was a possibility we wished to explore in the current study.

### The current study

The aim of this study was to extend our previous work [9] by assessing three commonly reported eye movement indices of attentional biases in SAD within a real-life social context for the first time. As such, we used a novel real-world setup where we monitored participants’ eye-movements using a mobile eye-tracker and analysed data for evidence of hypervigilance, avoidance and hyperscanning.

Participants (non-clinical) believed they were taking part in a real-world visual search study. Halfway through the study the experimenter left the room and the confederate, a young male entered. This created the potential for an interaction and the analysis was based on this period. However, we did not require the confederate to interact with the participants at all and discouraged him from entering into lengthy conversations with the participants, should they initiate them. This was because we were especially interested in the spontaneous behaviour which would naturally occur in an unstructured setting and which we felt was most comparable to much of everyday experience for university students.

Given the novelty of this design, developing appropriate hypotheses, which would ordinarily be based on a similar body of research, was difficult. Whilst the goal of the study was to determine if results from screen-based tasks of attention in social anxiety could be extended to real social scenarios, as outlined above, we expected there to be some stark differences in behaviour because unlike these studies, our participants were experiencing a genuine social event in real time. Therefore we took an exploratory approach and sought evidence for the three attentional bias indices most commonly reported in the eye tracking literature on social anxiety

1. Hypervigilance indicated by shorter time to first fixate the confederate and a higher proportion of first fixations on this region in participants with higher SA
2. Avoidance: operationalised as reduced dwell time and fewer fixations on the confederate over the whole period in higher SA participants
3. Hyperscanning: increased total scanpath length, reduced fixation count, and shorter mean fixation durations over the whole period in higher SA participants [19]

Additionally, in order to compare our data with those of other real-world social attention studies, we conducted an area of interest (AOI) analysis to determine the overall distribution of gaze within the scene.

Finally, we examined the influence of state anxiety on the above relationships.

## Method

### Participants

We conducted a power analysis using G*Power to determine sample size for this study. It was necessary to base the analysis on Howell and colleagues’ study [23], which was the most comparable in terms of aims and objectives to the present study, albeit utilising a screen-based task. This study reported a large (correlational) effect size (r = -.51) between scores on the Liebowitz Social Anxiety Scale (LSAS) [26] and average time to the “look zone” their primary eye tracking measure. Based on this, with an effect size of r = .51, α = .05 and power = .80 the projected sample size for the current study was N = 25.

We recruited thirty students between ages 18–39 years (*M* = 20.30, *SD* = 3.81; 8 males) from Bournemouth University who volunteered in exchange for course credit. All participants had normal or corrected-to-normal vision. SA was measured using the Liebowitz Social Anxiety Scale (LSAS) [26] (see below) with a sample mean of 53.07 (SD = 17.63).

Nine participants scored above 60 on LSAS, which is a cut-off for generalised SAD, the more severe presentation of the disorder whereas two participants scored below 30, which is a lower cut-off for performance -related SAD, where SAD is highly unlikely to be present [27].

### Materials and apparatus

The self-report Liebowitz Social Anxiety Scale (LSAS) [28] was used to measure trait SA. The scale comprises of 24 items measuring fear (on a scale from 0 = none to 3 = severe) and avoidance (on a scale from 0 = never to 3 = usually) experienced across different social and performance situations. Higher ratings indicate greater fear or avoidance. The LSAS has high internal consistency, with Chronbach’s alpha of .95, and good test-retest reliability at *r* = .82 [29]. Reliability analysis of our sample confirmed that LSAS Fear Scale (α = .88) is very good and the Avoidance scale is (α = .78) good.

State anxiety was assessed using the State-Trait Anxiety Inventory (STAI) [30] comprising 20 items assessing trait and 20 items assessing state anxiety. The items are rated on a 4-point scale (from “almost never” to “almost always”), where higher scores indicate greater anxiety. The inventory has high internal consistency, with Cronbach’s alpha ranging from .86 to.95, and adequate to good test-retest reliability, *r* coefficients range from .65 to .75 (37). Reliability analysis confirmed that STAI is (α = .88) very good in our sample. The trait anxiety element was not analysed.

The sham tasks included three visual search tasks: Where’s Wally [31], star cancellation from Behavioural Inattention Test [32] and organised shape cancellation from Cancellation Test [33]. Data were not analysed for this part of the study.

Eye-movements were recorded using the SMI Eye-Tracking Glasses 2 Wireless–ETG 2w (SensoMotoric Instruments) which has a binocular sampling rate of 120 Hz, with an average precision of 0.5-degree visual angle. The SMI ETG 2w smart recorder is based on a Samsung Galaxy Note 4.

### Procedure

Participants believed they would complete several pen and paper visual-search tasks as their eye-movements were recorded with a mobile eye tracker. The study took place in a Psychology department seminar room, where two seats were setup opposite one another, with the participant’s seat positioned one space to the left of the confederate’s seat. After reading the information sheet and signing the agreement form participants were assisted to put on the eye-tracking glasses, to allow time for adjustment, while completing the questionnaires.

Next, participants underwent a 3-point calibration procedure, suggested to be the best when recording at different distances according to manufacturers’ guidance. The researcher waited for 0-point calibration to automatically occur first, indicated by the change of colour (red to green) of the cursor. The landmarks used for 3-point calibration were located in a triangular pattern and participants had to look at the first landmark, while they did that, the screen was tapped to freeze the image and the crosshair displaying the landmark number was moved over to the landmark location in the image. This was repeated for the remaining 2 landmarks. Prior work using mobile eye tracking identified limitations in data quality due to increased movement and difficulty in calibration and validation procedures [34, 35]. Therefore, to ensure data quality, calibration was monitored throughout the study. If the cursor changed its colour to red from green, the calibration process would have been repeated. In addition, if participants could not achieve a successful calibration, they were unable to participate in the study, but this did not apply to any of our participants.

**Fig 1:**
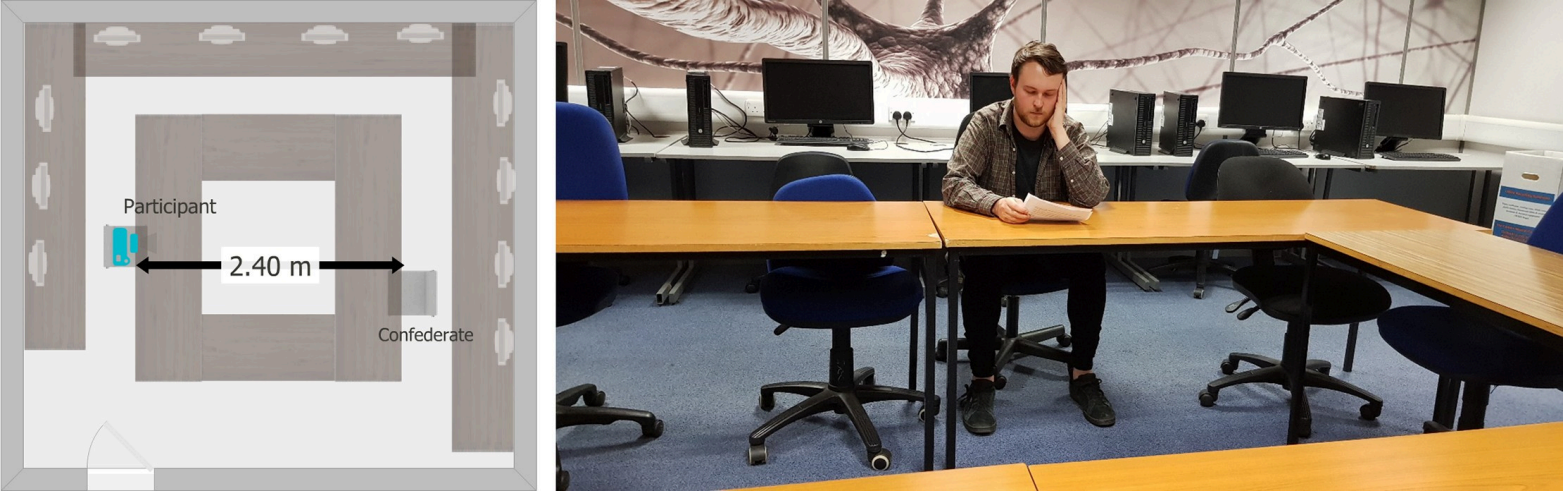
*a*. The room setup, showing location of the participant on the left and position of the confederate when seated, on the right. The door from which the confederate entered is shown at the bottom left. Figure is not drawn to scale. *b*. Reference image used to define participants’ gaze behaviour, showing the room layout. Image taken from the participants’ viewing position.

The eye-tracking recording then began, and participants completed the “Where’s Wally” task. The researcher left the room to collect the “forgotten” remaining visual search tasks. Participants were asked to remain in their seat, keeping the eye-tracking glasses on until the researcher returned. Participants did not engage in any tasks during researcher’s absence and all items were removed from participant’s immediate eyesight. Fifteen seconds after the researcher left, a Caucasian 25-year-old male confederate entered the room, briefly acknowledged the participant by nodding and gently smiling at them, and took a seat approximately 2.4m from the participant and began to complete questionnaires (see Fig 1 for room layout). The confederate was asked to keep his behaviour consistent across testing sessions and to not initiate a conversation with participants, however if a participant spoke to the confederate, he could respond. He also wore the same items of clothing for each session. When the researcher returned, participants completed the two cancellation tasks, following which the confederate left. Participants were asked if they had seen the confederate before and how well they knew him. If participants knew the confederate, they would have been excluded from the study to reduce the familiarity effect [36]. Finally, participants were debriefed about the true aim of the study and the need for deception. The study lasted 30 minutes.

The study was approved by Faculty of Science and Technology, Bournemouth University Research Ethics Committee (approval code 18153).

### Data handling

Eye-movement data was processed using BeGaze 3.7 (SensoMotoric Instruments, 2014). The analysis was based on the time between the confederate entering the room to the experimenter’s return *(M* = 61.09s, *SD* = 8.23). This critical interval time (CTI) was manually customised for each participant. Semantic gaze mapping was conducted manually to define where each participant was looking during the CTI. The reference image used to define the stimulus was a screenshot of the confederate as positioned during the experiment from the participants’ perspective (see Fig 1B). The image was divided into 3 areas of interest (AOIs): head, body, and background. The head and body interest areas included a small margin around each region to accommodate small errors eye tracking accuracy. The head AOI encompassed approximately 0.6° width of participants’ visual field (approximately 25cm diameter at a distance of 2.4m) and the body approximately 1.67° width (approximately 70cm at 2.4m distance) although this obviously altered slightly as the confederate moved. Any sample which did not fall within the head or body AOI was deemed to have fallen on the background AOI.

### Eye-movement measures

Dwell time percentage refers to the proportion of samples recorded for an AOI within the interest period [37]. Reduced dwell time to the social stimulus and increased dwell time to the background suggests avoidance of the social stimulus [5]. Fixation count refers to the number of fixations located in a specific AOI. Decreased fixation count to the social stimulus and increased fixation count to the background suggests avoidance [5]. Hypervigilance was measured by shorter time to first fixate the confederate and by longer first fixations towards him [5]. Duration to first fixation was measured manually from the scene camera footage recorded for each participant, from the first frame the confederate was visible on the screen.

In order to find evidence of hyperscanning, we examined several eye movement measures which are suggested by Chen et al. as possible markers of this monitoring strategy, The primary measure of hyperscanning is typically taken as a *longer total scanpath length* which is simple to extract from screen-based eye tracking datasets by summing all recorded saccade amplitudes, but this is less straightforward from mobile eye tacking data. Ostensibly this is because it can be difficult for algorithms to differentiate between eye movements and head movement. The event detection algorithm within the software for the mobile eye tracker used in this study (BeGaze 3.7; SensoMotoric Instruments) can accurately detect saccades with a true-positive reliability of 80% which is considerably higher than other commonly used algorithms calculating changes in sample peak velocities, according to the manufacturer [38]. That said, further criteria can be applied to the events labelled as saccades by the software which can further identify false-positives which may be artefacts of head movements. To this aim, scanpath lengths per participants were calculated by summing the saccade amplitudes of all saccades detected by the BeGaze event detection algorithm minus those with amplitudes outside of the range 1–20° visual angle [39] and peak velocities of 30–1000° /sec [40]. Events labelled as saccades by the algorithm which fell outside of these criteria are likely the result of head movement or are other artefacts, rather than eye movements.

In order to triangulate results from the saccade data, we additionally analysed total fixation count and mean fixation duration throughout the period of interest as both measures have been suggested to be related to scanpath length and attenuated measures of each are proposed to be associated with hyperscanning [19].

In addition, we analysed mean saccade amplitude and total saccade count across participants, in order to differentiate between those who had a long scanpath because of larger, but less numerous saccades and those with more frequent but smaller amplitude saccades, which might result in similar scanpath lengths, but a very different visual strategy.

### Data analysis

To assess the first three hypotheses, Pearson’s correlations were used with partial correlations used to account for the effect of state anxiety (state score from the STAI [28]. Tests of normality showed that all variables were normally distributed and hence suitable for this parametric test, except for the following: fixation count to head and body, dwell time to the head and background and time to first fixate the body. For correlations where one variable was not normally distributed both Pearson’s (*r*) and Spearman’s (*r*_*s*_) analyses were conducted and reported. Partial correlations are based on Pearson’s correlation regardless of distribution. All tests are two-tailed.

## Results

Once pre-processing had taken place, the eye movement data was checked for outliers (data points larger than 1.5 times the interquartile range for a given variable), by examining boxplots. The outlying data points (n = 19) were excluded for individual data points in eye-tracking measures [41], consisting of 4.22% of the total data.

Descriptive statistics for eye movement and self-report anxiety measures are shown in Table 1.

**Table 1:** Descriptive statistics for anxiety and eye-tracking measures.

	*Mean*	*SD*	*Range*
LSAS	53.07	17.63	18–93
State anxiety	35.5	8.24	21–53
Fixation duration (ms)	1088.64	452.19	191.20–2054.40
First fixation duration (ms)			
Head	140.24	56.67	82.80–248.90
Body	332.70	184.44	83.00–697.20
Fixation count			
Head	1.54	1.79	0.00–6.00
Body	8.00	9.11	0.00–33.00
Background	112.28	32.62	50.00–180.00
Total	127.30	39.74	51.00–211.00
Dwell time (%)			
Head	.60	.86	0.00–2.70
Body	4.12	4.92	0.00–15.80
Background	70.92	21.06	24.30–95.50
Time to first fixate (ms)			
Head	3896.14	3037.35	351.10–10867.30
Body	6018.85	5066.41	705.35–17234.15
`Saccade Amplitude			
(° visual angle)	5.49	1.23	3.21–8.05
Saccade Count	52.38	26.73	13.00–138.00
Scanpath Length			
(° visual angle)	295.30	171.47	41.70–790.00
Saccade rate (/ sec)*	.86	.44	.21–2.26

* Saccadic rate, calculated to compare to general eye tracking literature, is based on total saccades per participant divided by the mean trial length across participants (61.09 sec).

### Hypervigilance

No significant correlation was found between LSAS scores and time to first fixate the head *r* (16) = .101, *p* = .709 and the body *r* (21) = -.062, *p* = .790, (*r*_s_ (21) = -.144, *p* = .522). This remained the case when controlling for state anxiety using a partial correlation, *r* (13) = .088, *p* = .754, and the body *r* (18) = -.070, *p* = .7710

**Fig 2:**
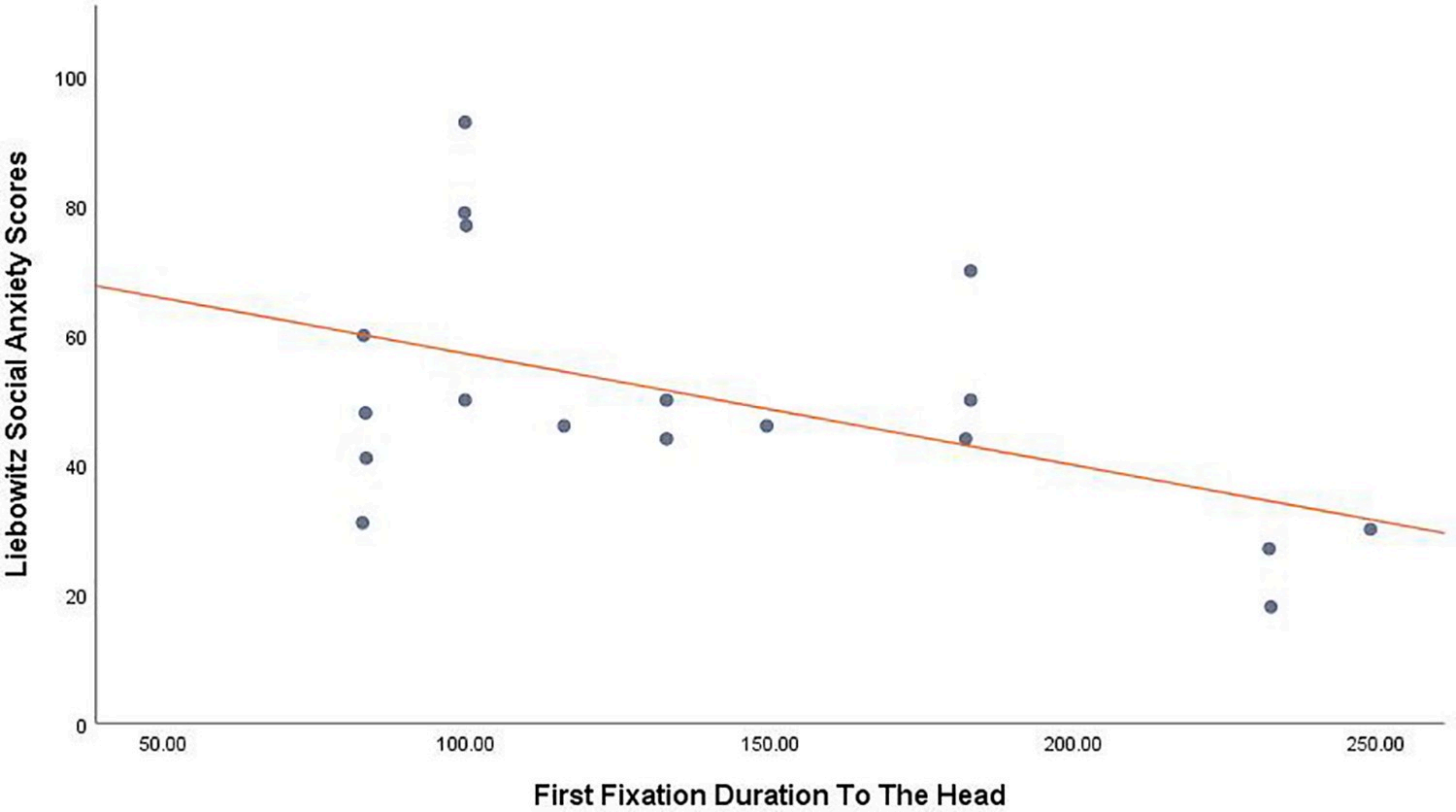
Scatterplot showing a correlation between first fixation duration to the head and SA traits.

A significant inverse relationship between LSAS scores and first fixation duration to the head was found, *r* (18) = -.502, *p* = .034 (*Fig 2*) (ignoring state anxiety) and *r* (15) = -.499, *p* = .041 (controlling for state anxiety).

There was no significant relationship between LSAS scores and first fixation duration to the body, *r* (23) = -.141, *p* = .521 which was also the case when controlling for state anxiety using a partial correlation *r* (20) = -.133, *p* =. 556

### Avoidance

There were no significant relationships between LSAS scores and dwell time to the head *r* (28) = -.073, *p* = .714 (*r*_s_ (28) = -.132, *p* = .502) the body *r* (30) = -.090, *p* = .637, and the background *r* (30) = -.231, *p* = .220 (*r*_s_ (30) = -.254, *p* = .175).

Partial correlations also revealed that when controlling for state anxiety, there was no significant relationship between the LSAS scores and dwell time to the head *r* (25) = -.063, *p* = .756, the body *r* (27) = -.090, *p* = .643, and the background *r* (27) = -.231, *p* = .229.

There was no significant relationship between the LSAS scores and fixation count to the head *r* (28) = .049, *p* = .805 (*r*_s_ (28) = -.055, *p* = .782), the body *r* (29) = -.075, *p* = .700 (*r*_s_ (21) = -.033, *p* = .865) and the background *r* (29) = -.258, *p* = .176. Partial correlations also showed that when controlling for state anxiety, there were still no significant relationships between the LSAS scores and fixation count to the head *r* (25) = .068, *p* = .743, the body *r* (26) = -.075, *p* = .704, and the background *r* (28) = -.258, *p* = .185

### Hyperscanning

**Fig 3:**
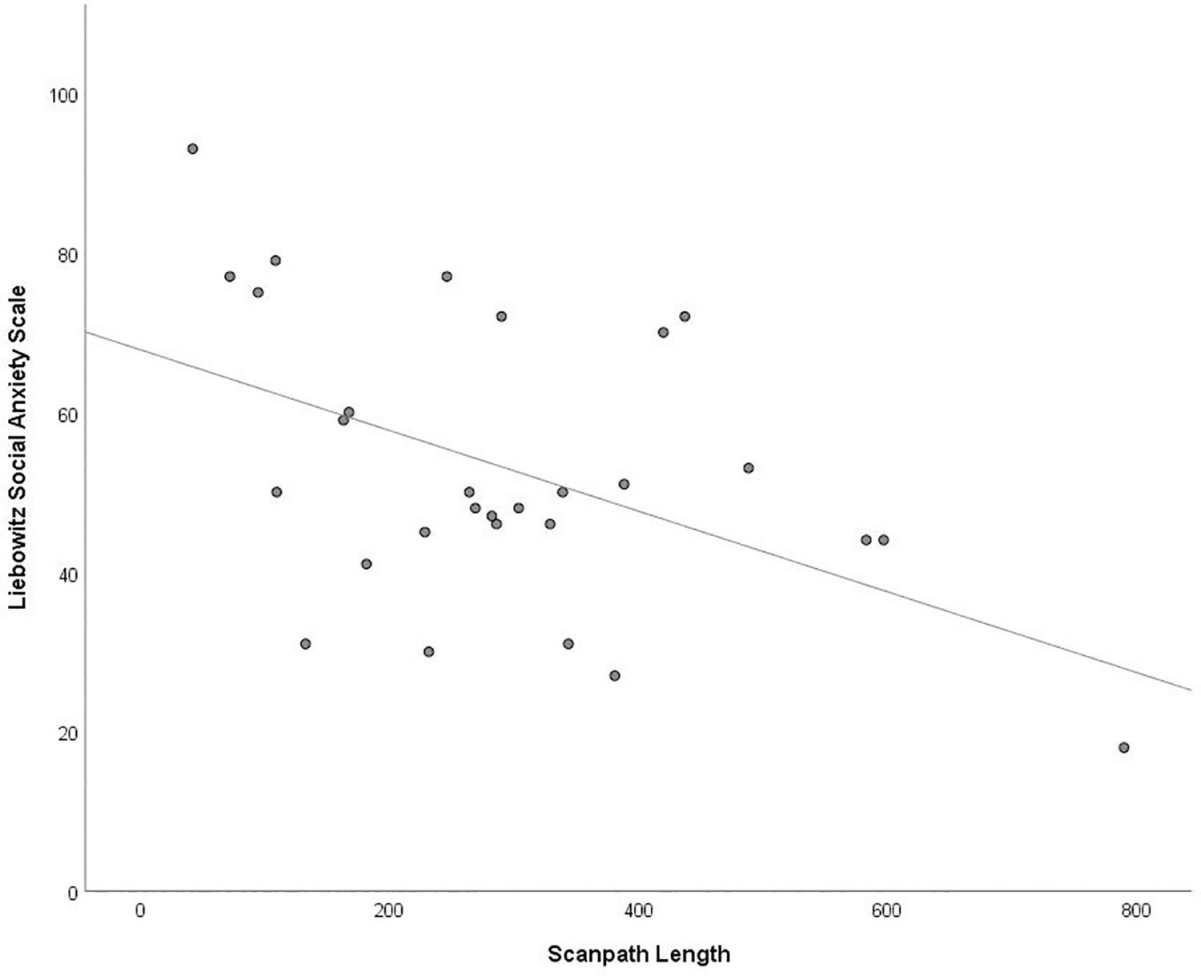
Scatterplot showing the significant negative correlation between scanpath length in degree of visual angle and score on the Liebowitz Social Anxiety Scale.

A strong inverse relationship was found between scanpath length and LSAS score, *r* (28) = -.483, *p* = .008 when ignoring state anxiety, and remained when state anxiety was controlled for *r* (26) = -.498, *p* = .007 (Fig 3).

A strong negative relationship was found between total saccade count and LSAS score, *r* (29) = -.511, *p* = .005 when ignoring state anxiety, which remained significant a when state anxiety was controlled for *r* (26) = -.516, *p* = .005. No relationship was found between mean saccade amplitude and LSAS score however, *r* (29) = -.176, *p* = .362 even when state anxiety was controlled for *r* (26) = -.160, *p* = .417

**Fig 4:**
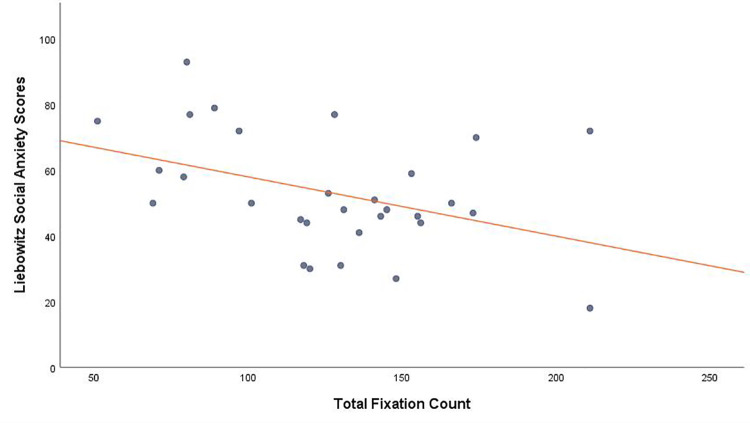
Scatterplot showing a negative correlation between total fixation count and SA traits.

There was a strong inverse relationship between LSAS scores and total fixation count, *r* (30) = -.407, *p* = .026 (without state anxiety) and *r* (27) = -.402, *p* = .030 (with state anxiety) (*Fig 4*). Saccade count, *r* (29) = .923, *p* < .001, and fixation count, *r* (29) = .697, *p* < .001 were both highly positively correlated with scanpath length, showing that more numerous saccades and fixations resulted in a longer scanpath overall. However mean fixation duration was not related to saccade count, *r* (29) = .067, *p* = .729, mean saccade amplitude, *r* (29) = .230, *p* = .229, scanpath length., *r* (29) = .134, *p* = .49, LSAS scores, *r* (30) = -.167, *p* = .378. or state anxiety, *r* (30) = .160, *p* = .397.

No relationship between LSAS scores and state anxiety existed, *r* (30) = -.073, *p* = .703. Correlation matrices for all correlations and partial correlations can be seen in Tables 2 and 3 respectively.

**Table 2:** Pearson correlation matrix for all eye movement and self-report measures.

	1	2	3	4	5	6	7	8	9	10	11	12	13	14	15	16	17	18
1. LSAS	1.000																	
2. STAI—state	-.073	1.000																
3. Mean fixation duration (ms)	-.167	.160	1.000															
4. First fixation duration to head (ms)	**-.502** ^*^	.082	.087	1.000														
5. First fixation duration to body (ms)	-.141	.135	**.604** ^**^	-.075	1.000													
6. Fixation count to head	.049	.241	**.458** ^*^	-.249	.157	1.000												
7. Fixation count to body	-.075	-.001	**.599** ^**^	.199	.355	.345	1.000											
8. Fixation count to background	-.258	.016	.192	.122	.119	.071	.251	1.000										
9. Total fixation count	**-.407** ^*^	.247	.276	.349	.168	.166	**.399** ^*^	**.938** ^**^	1.000									
10. Mean dwell time to head (ms)	-.073	-.073	**.461** ^*^	.119	.128	**.942** ^**^	**.572** ^**^	.129	.287	1.000								
11. Mean dwell time to body (ms)	-.090	.248	**.615** ^**^	.047	.312	**.486** ^**^	.963^**^	.204	**.405** ^*^	**.577** ^**^	1.000							
12. Mean dwell time to background (ms)	-.231	.029	**.394** ^*^	.093	.264	.030	-.007	**.585** ^**^	**.497** ^**^	-.094	-.128	1.000						
13. Time to first fixate head (ms)	.101	-.208	-.094	-.172	.004	.015	.141	-.211	-.198	.233	.115	-.373	1.000					
14. Time to first fixate body (ms)	-.062	-.103	-.314	-.200	-.072	.253	-.042	-.385	-.334	.248	-.088	**-.541** ^*^	**.598** ^*^	1.000				
15. Total scanpath length (° visual angle)	**-.483** ^**^	**.406** ^*^	.134	.461	-.003	.159	.145	**.572** ^**^	**.697** ^**^	.185	.122	**.385** ^*^	-.322	-.408	1.000			
16. Mean saccade amplitude (° visual angle)	-.176	**.402** ^*^	.230	.225	.070	.209	.259	-.001	.101	.096	.208	-.015	-.386	-.074	**.566** ^**^	1.000		
17. Total saccade count	**-.511** ^**^	.315	.067	.452	.036	.087	.093	**.785** ^**^	**.825** ^**^	.201	.087	**.447** ^*^	-.243	-.417	**.923** ^**^	.243	1.000	
18. Saccadic rate (saccades /sec)	**-.511** ^**^	.315	.067	.452	.036	.087	.093	**.785** ^**^	**.825** ^**^	.201	.087	**.447** ^*^	-.243	-.417	**.923** ^**^	.243	1.000^**^	1.000

LSAS = Liebowitz Social Anxiety Scale; STAI = State Trait Anxiety Inventory.

**. Correlation is significant at the .01 level (2-tailed).

*. Correlation is significant at the .05 level (2-tailed).

**Table 3:** Partial correlation matrix, controlling for state social anxiety scores from STAI for all eye movement measures against LSAS score.

	1	2	3	4	5	6	7	8	9	10	11	12	13	14	15	16	17
1. LSAS	1.000																
2. Mean fixation duration (ms)	-.158	1.000															
3. First fixation duration to head (ms)	-.499*	.075	1.000														
4. First fixation duration to body (ms)	-.133	**.596** **	-.087	1.000													
5. Fixation count to head	.068	**.438** *	-.278	.129	1.000												
6. Fixation count to body	-.075	**.607** **	.200	.358	.355	1.000											
7. Fixation count to background	-.258	.192	.121	.118	.069	.251	1.000										
8. Total fixation count	**-.402** *	.247	.340	.140	.113	**.412** *	**.964** **	1.000									
9. Mean dwell time to head (ms)	-.078	**.480** *	.125	.140	**.991** **	**.573** **	.131	.316	1.000								
10. Mean dwell time to body (ms)	-.074	**.602** **	.027	.290	.453	.994**	.207	.366	**.615** **	1.000							
11. Mean dwell time to background (ms)	-.229	**.394** *	.090	.263	.023	-.007	**.585** **	**.506** **	-.093	-.140	1.000						
12. Time to first fixate head (ms)	.088	-.063	-.159	.033	.068	.144	-.213	-.155	.224	.176	-.376	1.000					
13. Time to first fixate body (ms)	-.070	-.303	-.193	-.059	.287	-.042	-.385	-.321	.243	-.065	**-.541** **	.593	1.000				
14. Total scanpath length (° visual angle)	**-.498** **	.076	.469	-.064	.069	.159	**.619** **	**.674** **	.235	.024	**.409** **	-.266	-.403	1.000			
15. Mean saccade amplitude (° visual angle)	-.160	.184	.210	.018	.126	.283	-.008	.002	.137	.122	-.030	-.338	-.036	**.481** **	1.000		
16. Total saccade count	**-.516** **	.018	.450	-.007	.012	.098	**.822** **	**.812** **	.236	.010	**.461** *	-.191	-.408	**.917** **	.134	1.000	
17. Saccadic rate (saccades /sec)	**-.516** **	.018	.450	-.007	.012	.098	**.822** **	**.812** **	.236	.010	**.461** *	-.191	-.408	**.917** **	.134	1.000	1.000

LSAS = Liebowitz Social Anxiety Scale; STAI = State Trait Anxiety Inventory.

*. Correlation is significant at the .05 level (2-tailed).

**. Correlation is significant at the .01 level (2-tailed).

### AOI analysis

An ANOVA on dwell time and fixation count to the AOIs (head, body, background) showed significant main effects of dwell time (*F* (2, 58) = 293.85, η_p_^2^ = .910, *p* < .001) and fixation count (*F* (2, 58) = 289.70, η_p_^2^ = .909, *p* < .001) with participants allocating more dwell time and fixations to the background than the body with the fewest allocated to the head (all pairwise comparisons *p* < .001) (Table 1 for means).

## Discussion

This study employed a real-world eye-tracking setup to explore how SA influences attention allocation in a live social scenario. We predicted that 1) greater SA would be related to more avoidance of the confederate, 2) greater SA would be related to a hypervigilance to the confederate and 3) greater SA would be related to hyperscanning of the environment. We also expected that all participants regardless of SA levels would spend the greatest proportion of time gazing at the non-social aspects of the scene; Finally, we assessed the influence of state anxiety on these measures.

Contrary to our predictions, we found no relationship between dwell time and fixations to the confederate and SA traits. Previous research and models of SAD have proposed avoidance of social stimuli is a hallmark of the disorder. Rather, it appears that *all* participants, regardless of their SA status, avoided looking at the confederate. This is in stark contrast to a vast body of lab-based research overwhelmingly demonstrating that social stimuli attract attention above all other stimuli [42]. This discrepancy could be attributed to the fact that participants were physically present with the other social stimuli in the scene. In such real-world settings, when present in a confined space with a stranger, social norms of gaze behaviour are at play, particularly the norm of not indicating one’s excessive interest in the other, described elsewhere as “civil inattention” [43, 44]. Indeed, we found that on average participants made only 1.5 fixations to the confederate’s face during the whole recording. We have shown elsewhere that increasing the authenticity of the scene from pre-recorded to live and potentially interactive markedly reduces dwell time to the people within it [37, 45] and other researchers have demonstrated reduced social gaze during real-world viewing of others compared with viewing them onscreen [46, 47]. As gaze acts as both a channel and a signal for communication [48, 49], avoiding eye-contact when in the presence of a stranger acts as a socially safer response than gazing at them. Our results further support the interpretation that increased attention to faces may only occur when the participant views via the safe medium of the lab.

The consequence of this is that if *all* our participants were adhering to civil inattention, any additional avoidance by the higher SA participants may be masked. This brings us to the second hypothesis which was based on extensive experimental research [10, 50], that socially anxious participants would demonstrate hypervigilance towards the confederate. We found no evidence for this. Rather, and in line with our first hypothesis, highly SA participants’ first fixations to the confederate’s face were significantly *shorter* than those who were less anxious. We suggest this equates to an additional level of avoidance in the more SA participants over and above the more generic form of civil inattention found across the sample. It may at first seem counter-intuitive to suggest that fixating an object can in some sense represent avoidance of that object. However, as faces are such highly salient and rewarding stimuli, made additionally salient within our study as it was an animate, physically present human [51] it might not be surprising that even the highly SA participants could not fail to look at him to some extent. The fact that this fixation was shorter in the more anxious participants may reflect those participants’ unwillingness to maintain attention at this location despite being unable to avoid it entirely.

Based on observations from screen-based research, we predicted that higher SA participants would demonstrate hyperscanning, which is purported to be a hallmark of increased vigilance for threat, which could be indicated by several eye movement measures. However, we found that total scanpath length was significantly *shorter* in those with high SA which is completely at odds with the hyperscanning theory. In addition, contrary to previous suggestions that longer scanpaths should be related to fewer fixations, we found a strong relationship in the opposite direction which was equally strong for saccade frequency and scanpath length. So, although we found those with higher SA did execute fewer fixations than those who were less anxious, this was related to reduced rather than increased visual scanning. Fixation durations overall were not related to SA. In short, in our real-world paradigm, the more socially anxious participants engaged in less visual exploration than those who were less anxious in terms of fixations, saccades and total scanpath length.

To make sense of this unexpected finding, we can turn again to the concept “civil inattention” where, it is suggested, the social norm is to avoid looking directly at the other, to avoid signalling excessive interest when present with a stranger [43]. As eye contact is used as a non-verbal signal to initiate social interaction, not gazing at another signals to them that you do not wish to interact with them [37, 49] despite the overwhelming desire to do the opposite. This would necessitate a level of inhibitory control over one’s gaze.

If this is the case in non-anxious participants, what we have observed in our more socially anxious participants appears to be inhibition—not only of gaze towards the other—but of gaze: Full stop. Our more socially anxious participants committed fewer fixations, fewer saccades and an overall shorter scanpath throughout the whole trial period. Given in uncontrolled settings, humans typically make three to four saccades per second [52] this contrasts starkly with our sample who made on average .83 saccades per second in a range between .21 for the most anxious participants and 2.26 for the least socially anxious. This additional inhibition of eye movements in the higher SA participants could be described as hyper*- avoidance*, or *hypo*-scanning–in contrast to the hypervigilance and hyperscanning observed in even the most naturalistic of previously used tasks. These findings clearly demonstrate that in the case of gaze in social anxiety, the lab does not extrapolate to the real-world.

None of the significant results involving (trait) SA changed when accounting for state anxiety in our partial correlation analyses. Therefore, it would seem that state anxiety did not explain the relationships between SA and the gaze behaviour measures investigated here.

Our results are the first to chart eye-movements of participants with varying levels of SA within a real-world social setting. We have demonstrated that all participants avoided looking at the face but that the more SA participants looked for a shorter duration when first fixating him as well as executing fewer fixations, fewer saccades and a shorter scanpath overall. These findings suggest that a different viewing strategy is employed by participants higher in SA when in an unpredictable social setting, one which suggests an additional level of avoidance and inhibition over and above that shown by those lower in SA. Importantly, despite examining initial eye movement behaviour within the trial period, we found no evidence of hypervigilance reported by so many laboratory studies and which has informed recent theoretical models of SAD [4]. As such, our results challenge the conclusions drawn from this body of research which posits an increased vigilance for threat in this population. We propose that this hypervigilance may represent an artefact of experimental paradigms where the genuine social impact of the situation is greatly limited or non-existent. Instead, in our real-world setting, socially anxious individuals appear to adopt an inhibitory strategy over their eye movements, which not only results in fewer and shorter fixation to the individual present, but fewer fixations and saccades towards *anything* in their vicinity. One possible interpretation of this is that the higher SA participants were especially intent on avoiding social interaction due to their anxiety, and the most effective way to achieve this is to avoid committing any behaviour that might attract the confederate’s attention. Keeping the eyes especially still might be the only strategy available if one is already seated quietly at a desk.

A limitation of the current study is related to the size of the stimuli within the participants’ visual field in conjunction with the accuracy of the eye tracker. The head of the participant made up only around 0.6° of visual angle at a distance from the participant of 2.4m. With the accuracy of the eye tracker at 0.5°, we acknowledge that some samples may have assigned to an incorrect AOI. However, it is important to note that our main findings were not AOI-dependent and so any discrepancies resulting from misattribution of samples to AOIs would have been unlikely to have influenced the findings reported here. A further potential limitation was our non-clinical sample, which was made up of non-clinical, student participants. However, SA is considered to exist on a dimension of severity [53] and as such, this was a valid sample to utilise, especially given the novelty of the study. Although these participants had not been diagnosed with SAD, 6.6% of our sample scored below the lower clinical threshold on the LSAS and 30% scored above the higher threshold. However, it is acknowledged that our relatively small sample size (although greater than in the most comparable previous studies) could be extended in future studies. Having now demonstrated the viability of this paradigm, future research should investigate if these results can be replicated in a clinical sample. In addition, future studies could examine the impact of a more interactive component to the task such as presenting the participant with a task-related reason to engage with the confederate which is likely to evoke a distinct visual strategy given the role of eye gaze in communication [54].

Finally, for the purposes of maintaining control over confounding variables in the current study, the same male confederate was present with all participants, but future studies might investigate if sex of the confederate can modulate the attentional deployment in SA in a more naturalistic context, as has been suggested by previous research [55].

## Conclusion

Our results demonstrate that higher SA is related to attentional *hyper- avoidance* in a real-world unstructured social situation. This may represent a coping behaviour employed by socially anxious people to reduce anxiety and/ or avoid social interaction and goes against a vast body of experimental research demonstrating hypervigilance in SA in controlled, non-genuine experimental tasks. In addition, this study provides a novel paradigm for studying attention in authentic social settings which could be utilised across a spectrum of research domains implicating differences in attentional mechanisms.

## Supporting information

S1 Data Anonymised dataset containing self-report and eye movement measures. (SAV)

## References

[1] American Psychiatric Association. Diagnostic and Statistical Manual of Mental Disorders (DSM-5®). American Psychiatric Pub; 2013.

[2] Rapee RM, Heimberg RG. A cognitive-behavioral model of anxiety in social phobia. Behav Res Ther. 1997;35: 741–756. doi: 10.1016/s0005-7967(97)00022-3 9256517

[3] Clark DM, Wells A. A cognitive model of social phobia. Social phobia: Diagnosis, assessment, and treatment. New York, NY, US: Guilford Press; 1995. pp. 69–93.

[4] Wong QJJ, Rapee RM. The aetiology and maintenance of social anxiety disorder: A synthesis of complimentary theoretical models and formulation of a new integrated model. J Affect Disord. 2016;203: 84–100. doi: 10.1016/j.jad.2016.05.069 27280967

[5] Chen NTM, Clarke PJF. Gaze-Based Assessments of Vigilance and Avoidance in Social Anxiety: a Review. Curr Psychiatry Rep. 2017;19: 59. doi: 10.1007/s11920-017-0808-4 28726063

[6] Armstrong T, Olatunji BO. Eye tracking of attention in the affective disorders: A meta-analytic review and synthesis. Clin Psychol Rev. 2012;32: 704–723. doi: 10.1016/j.cpr.2012.09.004 23059623 PMC3556338

[7] Bantin T, Stevens S, Gerlach AL, Hermann C. What does the facial dot-probe task tell us about attentional processes in social anxiety? A systematic review. J Behav Ther Exp Psychiatry. 2016;50: 40–51. doi: 10.1016/j.jbtep.2015.04.009 26042381

[8] Waechter S, Nelson AL, Wright C, Hyatt A, Oakman J. Measuring Attentional Bias to Threat: Reliability of Dot Probe and Eye Movement Indices. Cogn Ther Res. 2014;38: 313–333. doi: 10.1007/s10608-013-9588-2

[9] Gregory NJ, Bolderston H, Antolin JV. Attention to faces and gaze-following in social anxiety: preliminary evidence from a naturalistic eye-tracking investigation. Cogn Emot. 2018; 1–12. doi: 10.1080/02699931.2018.1418197 30187816

[10] Klumpp H, Amir N. Examination of vigilance and disengagement of threat in social anxiety with a probe detection task. Anxiety Stress Coping. 2009;22: 283–296. doi: 10.1080/10615800802449602 19253172 PMC3712328

[11] Mogg K, Bradley B, Miles F, Dixon R. Brief Report Time Course of Attentional Bias for Threat Scenes: Testing the VigilanceAvoidance Hypothesis. Cogn Emot. 2004;18: 689–700.

[12] Gamble AL, Rapee RM. The time-course of attention to emotional faces in social phobia. J Behav Ther Exp Psychiatry. 2010;41: 39–44. doi: 10.1016/j.jbtep.2009.08.008 19781689

[13] Chen YP, Ehlers A, Clark DM, Mansell W. Patients with generalized social phobia direct their attention away from faces. Behav Res Ther. 2002;40: 677–687. doi: 10.1016/s0005-7967(01)00086-9 12051486

[14] Mansell W, Clark DM, Ehlers A, Chen Y-P. Social Anxiety and Attention away from Emotional Faces. Cogn Emot. 1999;13: 673–690. doi: 10.1080/026999399379032

[15] Garner M, Mogg K, Bradley BP. Orienting and maintenance of gaze to facial expressions in social anxiety. J Abnorm Psychol. 2006;115: 760–770. doi: 10.1037/0021-843X.115.4.760 17100533

[16] Singh JS, Capozzoli MC, Dodd MD, Hope DA. The Effects of Social Anxiety and State Anxiety on Visual Attention: Testing the Vigilance–Avoidance Hypothesis. Cogn Behav Ther. 2015;44: 377–388. doi: 10.1080/16506073.2015.1016447 25767901

[17] Heinrichs N, Hofmann SG. Information processing in social phobia: a critical review. Clin Psychol Rev. 2001;21: 751–770. doi: 10.1016/s0272-7358(00)00067-2 11434229

[18] Mogg K, Bradley BP, de Bono J, Painter M. Time course of attentional bias for threat information in non-clinical anxiety. Behav Res Ther. 1997;35: 297–303. doi: 10.1016/s0005-7967(96)00109-x 9134784

[19] Chen NTM, Thomas LM, Clarke PJF, Hickie IB, Guastella AJ. Hyperscanning and avoidance in social anxiety disorder: The visual scanpath during public speaking. Psychiatry Res. 2015;225: 667–672. doi: 10.1016/j.psychres.2014.11.025 25530414

[20] Horley K, Williams LM, Gonsalvez C, Gordon E. Social phobics do not see eye to eye: A visual scanpath study of emotional expression processing. J Anxiety Disord. 2003;17: 33–44. doi: 10.1016/s0887-6185(02)00180-9 12464287

[21] Horley K, Williams LM, Gonsalvez C, Gordon E. Face to face: visual scanpath evidence for abnormal processing of facial expressions in social phobia. Psychiatry Res. 2004;127: 43–53. doi: 10.1016/j.psychres.2004.02.016 15261704

[22] Wermes R, Lincoln TM, Helbig-Lang S. Anxious and alert? Hypervigilance in social anxiety disorder. Psychiatry Res. 2018;269: 740–745. doi: 10.1016/j.psychres.2018.08.086 30273899

[23] Howell AN, Zibulsky DA, Srivastav A, Weeks JW. Relations among Social Anxiety, Eye Contact Avoidance, State Anxiety, and Perception of Interaction Performance during a Live Conversation. Cogn Behav Ther. 2016;45: 111–122. doi: 10.1080/16506073.2015.1111932 26677735

[24] Quigley L, Nelson AL, Carriere J, Smilek D, Purdon C. The effects of trait and state anxiety on attention to emotional images: an eye-tracking study. Cogn Emot. 2012;26: 1390–1411. doi: 10.1080/02699931.2012.662892 22646929

[25] Kim H, Shin JE, Hong Y-J, Shin Y-B, Shin YS, Han K, et al. Aversive eye gaze during a speech in virtual environment in patients with social anxiety disorder. Aust N Z J Psychiatry. 2018;52: 279–285. doi: 10.1177/0004867417714335 28610445

[26] Liebowitz MR. Social Phobia. Mod Probl Pharmacopsychiatry. 1987;22: 141–173. doi: 10.1159/000414022 2885745

[27] Mennin DS, Fresco DM, Heimberg RG, Schneier FR, Davies SO, Liebowitz MR. Screening for social anxiety disorder in the clinical setting: using the Liebowitz Social Anxiety Scale. J Anxiety Disord. 2002;16: 661–673. doi: 10.1016/s0887-6185(02)00134-2 12405524

[28] Spielberger CD, Gorsuch RL, Lushene RE, Vagg PR, Jacobs PA. Manual for the State-Trait Anxiety Inventory. Palo Alto: Consulting Psychologists Press; 1983.

[29] Baker SL, Heinrichs N, Kim H-J, Hofmann SG. The Liebowitz social anxiety scale as a self-report instrument: a preliminary psychometric analysis. Behav Res Ther. 2002;40: 701–715. doi: 10.1016/s0005-7967(01)00060-2 12051488

[30] Spielberger CD, Gorsuch RL, Lushene RE. Manual for the State-Trait Anxiety Inventory. 1970 [cited 23 Jan 2020]. Available: http://ubir.buffalo.edu/xmlui/handle/10477/2895

[31] Handford M. Where’s Wally? 30th Anniversary Edition with a Bonus Scene. London: Walker Books; 2017.

[32] Wilson B, Cockburn J, Halligan P. Development of a behavioral test of visuospatial neglect. Arch Phys Med Rehabil. 1987;68: 98–102. 3813864

[33] Stone SP, Halligan PW, Wilson B, Greenwood RJ, Marshall JC. Performance of age-matched controls on a battery of visuo-spatial neglect tests. J Neurol Neurosurg Psychiatry. 1991;54: 341–344. doi: 10.1136/jnnp.54.4.341 2056320 PMC488490

[34] Fu X, Nelson EE, Borge M, Buss KA, Pérez-Edgar K. Stationary and ambulatory attention patterns are differentially associated with early temperamental risk for socioemotional problems: Preliminary evidence from a multimodal eye-tracking investigation. Dev Psychopathol. 2019;31: 971–988. doi: 10.1017/S0954579419000427 31097053 PMC6935016

[35] Fu X, Pérez-Edgar K. Threat-related attention bias in socioemotional development: A critical review and methodological considerations. Dev Rev. 2019;51: 31–57. doi: 10.1016/j.dr.2018.11.002 32205901 PMC7088448

[36] Cordell DM, McGahan JR. Mutual gaze duration as a function of length of conversation in male-female dyads. Psychol Rep. 2004;94: 109–114. doi: 10.2466/pr0.94.1.109-114 15077754

[37] Gregory N, Antolin JV. Does Social Presence or the Potential for Interaction reduce Social Gaze in Online Social Scenarios? Introducing the “Live Lab” paradigm. Q J Exp Psychol. 2018 [cited 3 May 2018]. doi: 10.1177/1747021818772812 29649946

[38] Sensomotoric Instruments. Technical Report SMI Event Detection For Mobile Eye Trackers. n.d.

[39] Gilchrist I. Chpater 5: Saccades. In: Liversedge SP, Gilchrist I, Everling S, editors. The Oxford Handbook of Eye Movements. OUP Oxford; 2011.

[40] Holmqvist K, Nyström M, Andersson R, Dewhurst R, Jarodzka H, Weijer J van de. Eye Tracking: A comprehensive guide to methods and measures. OUP Oxford; 2011.

[41] Field A. DISCOVERING STATISTICS USING IBM SPSS STATISTICS. 4th edition. Sage Publications; 2013.

[42] Flechsenhar AF, Gamer M. Top-down influence on gaze patterns in the presence of social features. PLOS ONE. 2017;12: e0183799. doi: 10.1371/journal.pone.0183799 28837673 PMC5570331

[43] Goffman E. Behavior in Public Places. Simon and Schuster; 1963.

[44] Zuckerman M, Miserandino M, Bernieri F. Civil Inattention Exists—in Elevators. Pers Soc Psychol Bull. 1983;9: 578–586. doi: 10.1177/0146167283094007

[45] Gregory NJ, Lόpez B, Graham G, Marshman P, Bate S, Kargas N. Reduced Gaze Following and Attention to Heads when Viewing a “Live” Social Scene. PLoS ONE. 2015;10: e0121792. doi: 10.1371/journal.pone.0121792 25853239 PMC4390321

[46] Foulsham T, Walker E, Kingstone A. The where, what and when of gaze allocation in the lab and the natural environment. Vision Res. 2011;51: 1920–1931. doi: 10.1016/j.visres.2011.07.002 21784095

[47] Laidlaw KEW, Foulsham T, Kuhn G, Kingstone A. Potential social interactions are important to social attention. Proc Natl Acad Sci. 2011;108: 5548–5553. doi: 10.1073/pnas.1017022108 21436052 PMC3078350

[48] Argyle M, Ingham R, Alkema F, McCallin M. The Different Functions of Gaze. 1973. doi: 10.1515/semi.1973.7.1.19

[49] Gobel MS, Kim HS, Richardson DC. The dual function of social gaze. Cognition. 2015;136: 359–364. doi: 10.1016/j.cognition.2014.11.040 25540833

[50] Mogg K, Philippot P, Bradley BP. Selective attention to angry faces in clinical social phobia. J Abnorm Psychol. 2004;113: 160–165. doi: 10.1037/0021-843X.113.1.160 14992669

[51] Klein J, Shepherd S, Platt M. Social attention and the brain. Curr Biol. 2009;19: R958–R962. doi: 10.1016/j.cub.2009.08.010 19889376 PMC3387539

[52] Findlay JM, Gilchrist ID. Active vision the psychology of looking and seeing. Oxford; New York: Oxford University Press; 2003.

[53] Ruscio AM. The Latent Structure of Social Anxiety Disorder: Consequences of Shifting to a Dimensional Diagnosis. J Abnorm Psychol. 2010;119: 662–671. doi: 10.1037/a0019341 20853918 PMC2991527

[54] Risko EF, Richardson DC, Kingstone A. Breaking the Fourth Wall of Cognitive Science Real-World Social Attention and the Dual Function of Gaze. Curr Dir Psychol Sci. 2016;25: 70–74. doi: 10.1177/0963721415617806

[55] Wieser MJ, Pauli P, Weyers P, Alpers GW, Mühlberger A. Fear of negative evaluation and the hypervigilance-avoidance hypothesis: an eye-tracking study. J Neural Transm. 2009;116: 717–723. doi: 10.1007/s00702-008-0101-0 18690409

